# Effects of Kynurenine Pathway Metabolites on Intracellular NAD^+^ Synthesis and Cell Death in Human Primary Astrocytes and Neurons

**DOI:** 10.4137/ijtr.s2318

**Published:** 2009-04-03

**Authors:** Nady Braidy, Ross Grant, Bruce J Brew, Seray Adams, Tharusha Jayasena, Gilles J. Guillemin

**Affiliations:** 1 University of New South Wales, Faculty of Medicine, Sydney, Australia; 2 Australasian Research Institute, Sydney Adventist Hospital, Sydney, Australia; 3 St. Vincent’s Centre for Applied Medical Research, Sydney, Australia; 4 Department of Neurology, St. Vincent’s Hospital, Sydney, Australia; 5 Bioanalytical Mass Spectrometry Facility, University of New South Wales, Sydney, Australia

## Abstract

The kynurenine pathway (KP) is a major route of L-tryptophan catabolism resulting in the production of the essential pyridine nucleotide nicotinamide adenine dinucleotide, (NAD^+^). Up-regulation of the KP during inflammation leads to the release of a number of biologically active metabolites into the brain. We hypothesised that while some of the extracellular KP metabolites may be beneficial for intracellular NAD^+^ synthesis and cell survival at physiological concentrations, they may contribute to neuronal and astroglial dysfunction and cell death at pathophysiological concentrations. In this study, we found that treatment of human primary neurons and astrocytes with 3-hydroxyanthranilic acid (3-HAA), 3-hydroxykynurenine (3-HK), quinolinic acid (QUIN), and picolinic acid (PIC) at concentrations below 100 nM significantly increased intracellular NAD^+^ levels compared to non-treated cells. However, a dose dependent decrease in intracellular NAD^+^ levels and increased extracellular LDH activity was observed in human astrocytes and neurons treated with 3-HAA, 3-HK, QUIN and PIC at concentrations >100 nM and kynurenine (KYN), at concentrations above 1 μM. Intracellular NAD^+^ levels were unchanged in the presence of the neuroprotectant, kynurenic acid (KYNA), and a dose dependent increase in intracellular NAD^+^ levels was observed for TRP up to 1 mM. While anthranilic acid (AA) increased intracellular NAD^+^ levels at concentration below 10 μM in astrocytes. NAD^+^ depletion and cell death was observed in AA treated neurons at concentrations above 500 nM. Therefore, the differing responses of astrocytes and neurons to an increase in KP metabolites should be considered when assessing KP toxicity during neuroinflammation.

## Introduction

Tryptophan (TRP) catabolism via the kynurenine pathway (KP) represents the major pathway for the synthesis of nicotinamide adenine dinucleotide (NAD^+^).^1^ Essential NAD^+^dependent reactions can be divided into three main categories:^2^ (1) NAD^+^ is an important contributor to energy (ATP) production;^3^ (2) NAD^+^ serves as a cofactor for NAD glycohydrolases involved in intracellular calcium regulation;^4^,^5^ (3) NAD^+^ is a substrate for the family of DNA nick sensing poly(ADP-ribose) polymerases (PARP)^6^–^8^ and the class III histone deacetylases known as sirtuins.^9^,^10^ NAD^+^ levels are extremely volatile and can be significantly reduced under conditions of excessive PARP-1 activation caused by oxidative damage to DNA, and during mitosis.^11^ Thus, continuous biosynthesis of NAD^+^ is vital to the maintenance and ongoing cell viability of all cells.^12^

**Figure 1.**
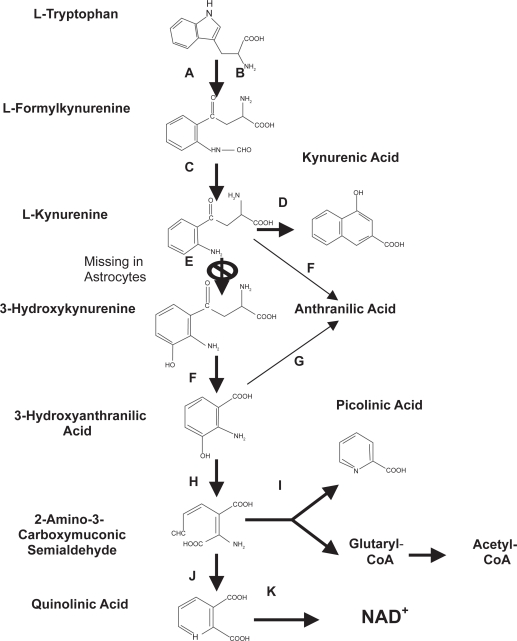
The Kynurenine Pathway of Tryptophan Degradation. **A**) Indoleamine 2,3-dioxygenase (IDO); **B**) Tryptophan 2,3 dioxygenase (TDO) **C**) Kynurenine Formylase; **D**) Kynurenine-Amino Transferase; **E**) Kynurenine 3Hydroxylase; **F**) Kynureninase; **G**) Non-specific hydroxylation; **H**) 3-Hydroxyanthranilic Acid Oxidase; **I**) Picolinic Carboxylase **J**) Non-enzymatic cyclisation; **K**) Quinolinic Acid Phosphoribosyltransferase.

The KP is the principal route of L-tryptophan catabolism, resulting in the production of NAD^+^ (Fig. 1). Over-activation of the KP has been implicated in the pathogenesis of several neurological disorders including Huntington’s disease (HD), Alzheimer’s disease (AD), and the acquired immunodeficiency syndrome (AIDS)-dementia complex.^13^–^17^ The pathway is regulated by the immune-factor responsive enzyme indoleamine-2,3-dioxygenase (IDO) in most cells and by tryptophan-2,3 dioxygenase (TDO) in the liver which is modulated by tryptophan and glucocorticoids.^18^,^19^

Several intermediate products of the KP are known to be neurotoxic. Among them, the N-methyl-D-aspartate (NMDA) receptor agonist and neurotoxin, quinolinic acid (QUIN) is likely to be most important in terms of biological activity.^15^ Anthranilic acid (AA), 3-hydroxyanthranilic acid (3-HAA), and 3-hydroxykynurenine (3-HK) have been shown to generate free radicals leading to neuronal damage similar to QUIN.^15^ The early upstream KP metabolite kynurenic acid (KYNA), has been shown to antagonise the neurotoxic effects of QUIN and glutamate-mediated NMDA receptor activation.^20^,^21^ The downstream metabolite picolinic acid (PIC) is an endogenous metal chelator within the brain^22^,^23^ that displays some protection against QUIN induced toxicity and posesses immune regulatory activity.^24^,^25^

Given the significance of intracellular NAD^+^ levels for the maintenance of total cell integrity and cell viability, we used primary monocultures of human neurons and astrocytes treated with physiological and pathophysiological concentrations of TRP, KYN, KYNA, AA, 3-HAA, 3-HK, PIC, and QUIN respectively (0.1–100 μM). Intracellular NAD^+^ levels were measured using the thiazolyl blue microcycling assay. The effect of KP metabolites on cell viability was determined by measuring the release of lactate dehydrogenase into the extracellular medium.

## Materials and Methods

### Reagents and chemicals

Dulbecco’s phosphate buffer solution (DBPS) and all other cell culture media and supplements were from Invitrogen (Melbourne, Australia) unless otherwise stated. Nicotinamide, bicine, β-nicotinamide adenine dinucleotide reduced form (β-NADH), 3-[-4,5-dimethylthiazol-2-yl]-2,5-diphenyl tetrazolium bromide (MTT), alcohol dehydrogenase (ADH), sodium pyruvate, TRIS, γ-globulins, L-tryptophan (TRP), kynurenine (KYN), kynurenic acid (KYNA), anthranilic acid (AA), 3-hydroxyanthranilic acid (3-HAA), 3-hydroxykynurenine (3-HK), picolinic acid (PIC), and quinolinic acid (QUIN) were obtained from Sigma-Aldrich (Castle-Hill, Australia). Phenazine methosulfate (PMS) was obtained from ICN Biochemicals (Ohio, U.S.A). Bradford reagent was obtained from BioRad, Hercules (CA, U.S.A).

### Cell cultures

Human foetal brains were obtained from 16–19 week old foetuses collected following therapeutic termination with informed consent. Mixed brain cultures were prepared and maintained using a protocol previously described by Guillemin et al.^26^ Astrocytes and neurons were prepared from the mixed brain cell cultures, and maintained using a protocol previously described by Guillemin et al.^27^

### Primary brain cells and KP metabolite culture treatments

Human primary astrocytes and neurons were incubated with various concentrations of the KP metabolites (0.1–100 μM) for 24 hours. Experiments were performed with primary cultures derived from three different human foetal brains with each individual preparation tested in triplicate.

### NAD(H) Microcycling assay for the measurement of intracellular NAD^+^ concentrations

Intracellular NAD^+^ concentration following 24 hour incubation with the desired concentrations of KP metabolites were measured spectrophotometrically using the thiazolyl blue microcycling assay established by Bernofsky and Swan^28^ adapted for 96 well plate format by Grant and Kapoor.^29^

### Extracellular LDH activity as a measurement for cytotoxicity

The release of lactate dehydrogenase (LDH) into culture supernatant correlates with the amount of cell death and membrane damage, providing an accurate measure of cellular toxicity. LDH activity following 24 hour incubation with the desired concentrations of KP metabolites was assayed using a standard spectrophotometric technique described by Koh and Choi.^30^

### Bradford protein assay for the quantification of total protein

NAD^+^ concentration and extracellular LDH activity were adjusted for variations in cell number using the Bradford protein assay described by Bradford.^31^

### Data analysis

Results obtained are presented as the means ± the standard error of measurement (SEM). Significant differences between results were verified using the two-tailed t-test with equal variance. Differences between treatment groups were considered significant if p was less than 0.05 (p < 0.05).

## Results

### 

#### Effect of extracellular KP metabolites on intracellular NAD^+^ concentrations in human astrocytes and neurons

**Figure 2.**
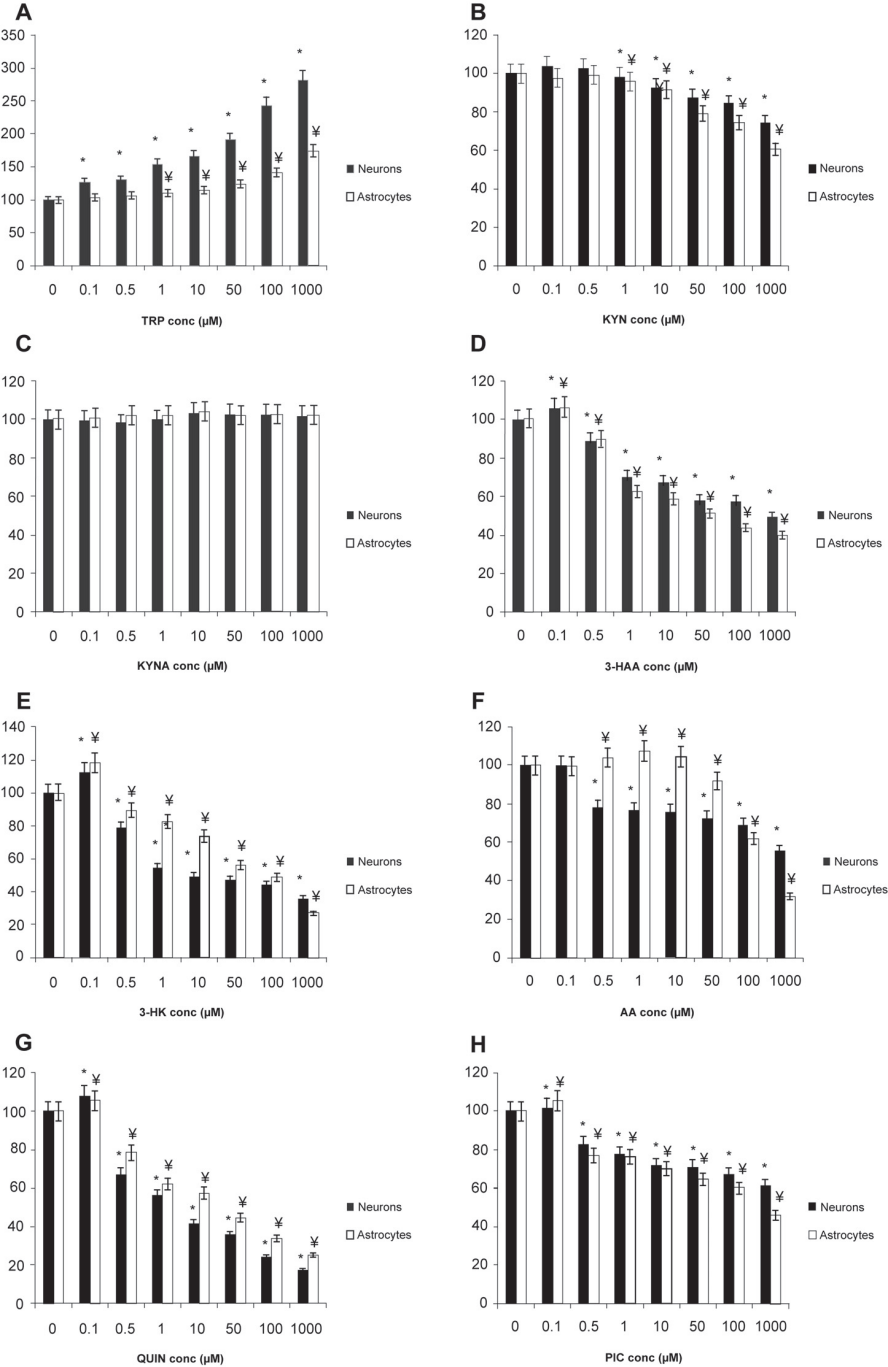
The effect of KP metabolites on intracellular NAD^+^ levels in human neurons and astrocytes. (Control = 0 μM treatment for each metabolite and cell type) **A**) The effect of TRP (1–1000 μM) on intracellular NAD^+^ in human neurons and astrocytes after 24 hours. *p < 0.05 compared to control in human neurons. ¥p < 0.05 compared to control in human astrocytes. **B**) The effect of KYN (1–1000 μ M) on intracellular NAD^+^ in human neurons and astrocytes after 24 hours. *p < 0.05 compared to control in human neurons. ¥p < 0.05 compared to control in human astrocytes. **C**) The effect of KYNA (1–1000 μM) on intracellular NAD^+^ in human neurons and astrocytes after 24 hours. *p < 0.05 compared to control in human neurons. ¥p < 0.05 compared to control in human astrocytes. **D**) The effect of 3-HAA (1–1000 μM) on intracellular NAD^+^ in human neurons and astrocytes after 24 hours. *p < 0.05 compared to control in human neurons. ¥p < 0.05 compared to control in human astrocytes. **E**) The effect of 3HK (1–1000 μM) on intracellular NAD^+^ in human neurons and astrocytes after 24 hours. *p < 0.05 compared to control in human neurons. ¥p < 0.05 compared to control in human astrocytes. **F**) The effect of AA (1–1000 μ M) on intracellular NAD^+^ in human neurons and astrocytes after 24 hours. *p < 0.05 compared to control in human neurons. ¥p < 0.05 compared to control in human astrocytes. **G**) The effect of QUIN (1–1000 μM) on intracellular NAD^+^ in human neurons and astrocytes after 24 hours. *p < 0.05 compared control in human neurons. ¥p < 0.05 compared to control in human astrocytes. **H**) The effect of PIC (1–1000 μM) on intracellular NAD^+^ in human neurons and astrocytes after 24 hours. *p < 0.05 compared to control in human neurons. ¥p < 0.05 compared to control in human astrocytes. (n = 3 for each treatment group).

TRP induced a dose-dependent increase in intracellular NAD^+^ levels in both human neurons (at >100 nM TRP) and astrocytes (at >500 nM TRP), (Fig. 2). We found that 3-HAA, 3-HK, QUIN and PIC significantly increased intracellular NAD^+^ levels at a low concentration of 100 nM but substantially decreased NAD^+^ levels at higher concentrations (Fig. 2) in both cell types. Treatment with KYNA had no significant effect on intracellular NAD^+^ activity in either neuronal or astroglial cultures (Fig. 2). A dose-dependent decrease in intracellular NAD^+^ levels was observed in KYN treated astrocytes and neurons at concentrations above 1 μM. AA increased intracellular NAD^+^ levels at concentrations below 50 μM in human astrocytes. However in human neurons NAD^+^ depletion was observed at AA concentrations ≥500 nM for neurons and ≥100 μM for astrocytes (Fig. 2).

#### Effect of extracellular KP metabolites on extracellular LDH activity in human astrocytes and neurons

**Figure 3.**
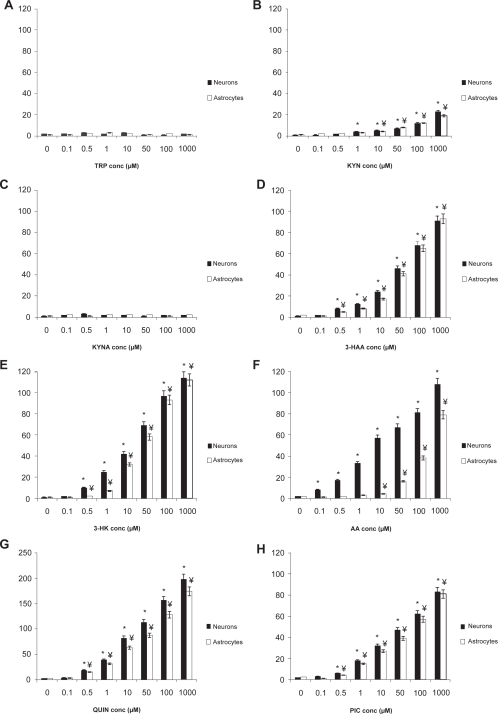
Effect of KP metabolites on extracellular LDH activity in human astrocytes and neurons (Control = 0 μM treatment for each metabolite and cell type). **A**) The effect of TRP (1–1000 μM) on extracellular LDH activity in human neurons and astrocytes after 24 hours. *p < 0.05 compared to control in human neurons. ¥p < 0.05 compared to control in human astrocytes. **B**) The effect of KYN (1–1000 μM) on extracellular LDH activity in human neurons and astrocytes after 24 hours. *p < 0.05 compared to control in human neurons. ¥p < 0.05 compared to control in human astrocytes. **C**) The effect of KYNA (1–1000 μM) on extracellular LDH activity in human neurons and astrocytes after 24 hours. *p < 0.05 compared to control in human neurons. ¥p < 0.05 compared to control in human astrocytes. **D**) The effect of 3-HAA (1–1000 μM) on extracellular LDH activity in human neurons and astrocytes after 24 hours. **p < 0.05 compared to control in human neurons (n = 3 for each treatment group). ¥p < 0.05 compared to control in human astrocytes. **E**) The effect of 3-HK (1–1000 μM) on extracellular LDH activity in human neurons and astrocytes after 24 hours. *p < 0.05 compared to control in human neurons. ¥p < 0.05 compared to control in human astrocytes. **F**) The effect of AA (1–1000 μM) on extracellular LDH activity in human neurons and astrocytes after 24 hours. *p < 0.05 compared to control in human neurons. ¥p < 0.05 compared to control in human astrocytes. **G**) The effect of QUIN (1–1000 μM) on extracellular LDH activity in human neurons and astrocytes after 24 hours. *p < 0.05 compared to control in human neurons. ¥p < 0.05 compared to control in human astrocytes. **H**) The effect of PIC (1–1000 μM) on extracellular LDH activity in human neurons and astrocytes after 24 hours. *p < 0.05 compared to control in human neurons. ¥p < 0.05 compared to control in human astrocytes (n = 3 for each treatment group).

Consistent with the results obtained for intracellular NAD^+^ levels, no significant change was observed in extracellular LDH activity for both astrocyte and neuronal cultures treated with TRP or KYNA up to 1 mM (Fig. 3). However treatment with 3-HAA, 3-HK, QUIN and PIC increased extracellular LDH activity at concentrations above 100 nM (Fig. 3) in both cell types. A dose-dependent increase in extracellular LDH activity was observed in astrocytes and neurons treated with KYN at concentrations above 1 μM. However the magnitude of LDH release following KYN treatment was significantly less than that observed for any of the other toxic metabolites. LDH activity was also increased for cells treated with AA at concentrations above 100 nM in astrocytes and at or above 100 nM in neurons (Fig. 3).

## Discussion

Neurodegenerative diseases are often characterised by a loss of neuronal cells in specific regions of the brain. Given the importance of intracellular NAD^+^ levels for maintaining overall cellular integrity and function, it is conceivable that reduced NAD^+^ levels are a potential pathogenic mechanism for neuronal and astroglial cell death.^11^,^8^ TRP has been used as a supplement for some years in the United States before being removed due to an outbreak of the lethal autoimmune disease, eosinophilia-myalgia syndrome (EMS) resulting in 36 deaths.^11^ Large doses of TRP can induce the build-up of selected white blood cells leading to EMS. Since the KP is a major regulator of the immune response,^32^ the toxic effect may be due to inhibition of normal tolerogenic cell T-cell death following IDO induction.^11^

In this study, we found that TRP supplementation produced a dose-dependent increase in intracellular NAD^+^ levels in human astrocytes and neurons after 24 hours (Fig. 2). The physiological concentration of TRP is human plasma is estimated to be 40–90 μM.^33^,^34^ Lower serum concentrations have been observed in several disorders including depression and anxiety,^35^ rheumatoid arthritis^36^ and following infection with HIV.^33^ TRP supplementation has been previously shown to be beneficial in several neurological conditions, including insomnia and depression, since TRP can be used for the synthesis of serotonin, melatonin and NAD^+^.^11^,^19^ Moreover, under conditions of TRP depletion, supplementation with TRP down-regulates enzymes directing TRP to non-NAD^+^ dependent pathways,^19^ suggesting a shift of all available TRP catabolism to NAD^+^ synthesis.^37^ However, it is clear that excessive TRP supplementation would aggravate or induce autoimmune disease.^11^

The primary metabolite of TRP, N-formylkynurenine can be rapidly converted to KYN by the enzyme arylamine formamidase.^38^ KYN (its physiological concentration is 1 μM) is converted into several neurotoxic metabolites such as 3-HK and QUIN in unstimulated human brain cells.^39^,^14^ Our data indicates that KYN causes intracellular NAD^+^ depletion and reduced cell viability at greater than physiological concentrations (Figs. 2 and 3). Human astrocytes and microglial cells demonstrate rapid cellular uptake of KYN. KYN has been shown to increase QUIN production and KP enzyme expression in human macrophages.^27^ The enhanced generation of QUIN may account for the reduced NAD^+^ levels and increased cytotoxicity observed in human neurons and astrocytes at pathophysiological concentrations (Figs. 2 and 3).

KYN and the resulting metabolite, 3-HK can be converted to AA and 3-HAA by kynureninase.^15^ These metabolites also provide additional substrate for QUIN formation.^40^ The levels of 3-HK, AA and 3-HAA are significantly increased in the CSF of patients with HD.^41^ Elevated levels have also been reported in HIV cases associated with dementia, infantile spasms, and hepatic encephalopathy.^40^ While 3-HK, AA, and 3-HAA appear less neurotoxic than QUIN, these KP metabolites have been previously shown to promote neuronal damage largely through free radical formation but not NMDA receptor activation.^15^ Under normal conditions, the serum concentration of 3-HK, 3-HAA and AA has been found to be 383 nM,^42^ 24 nM^35^ and 21 nM,^34^ respectively. In this study, we found that 3-HK, AA and 3-HAA supplementation resulted in neuronal and astroglial NAD^+^ depletion and cell death in 24 hour cultures at micromolar concentrations (Fig. 1 and Fig. 2). These results are consistent with a previous study showing that 3-HK, 3-HAA, and AA also induced a time-and dose-dependent increase in cell death at micromolar concentrations (1–100 μM).^41^ These authors reported that the accompanying cell death was significantly reduced by co-treatment with catalase, suggesting that the neurotoxic effects of 3-HK may be mediated by increased hydrogen peroxide.^41^ 3-HK can be converted to quinoneimines that can generate pro-oxidant intermediates, such as hydrogen peroxide, organic and hydroxyl radicals, during processes of autoxidation.^43^,^44^ Similarly, 3-HK has been shown to potentiate QUIN toxicity in rats, and the cytotoxic effect can be prevented using free radical scavengers.^45^

Although 3-HK and 3-HAA have been shown to be cytotoxic at high concentrations, physiological concentrations of 100 nM increased intracellular NAD^+^ levels by 18 and 12 per cent respectively, with no detectable effect on extracellular LDH activity (Figs. 1 and Fig. 2). The contribution of 3-HK and 3-HAA at these physiological concentrations to neuronal and astroglial cytotoxicity is uncertain as micromolar concentrations appear to produce neurotoxicity *in-vitro.*^41^,^45^

The observation that addition of these KP metabolites can serve as substrate for NAD^+^ synthesis at low concentrations (100–500 nM) implies that under normal conditions, NAD^+^ levels may be dependent on substrate availability.^29^ We propose that astroglial and neuronal KP enzymes downstream of QUIN may become saturated in the presence of excess extracellular KP metabolites leading to an accumulation of QUIN. Indeed, we have previously demonstrated that exogenous 3-HAA substantially increases QUIN synthesis in human foetal astrocytes to neurotoxic levels implying saturation of downstream enzymes involved in catabolism of QUIN to its essential metabolite, NAD^+^.^27^ Similarly, Blight et al^46^ observed that treatment of 4-chloro-3-hydroxyanthranilate, a synthetic inhibitor of 3-hydroxyanthranilic acid oxidase, was able to reduce QUIN production and functional deficits following experimental spinal cord injury in guinea pigs.

On the other hand, our results indicate a clear dichotomy between exogenous AA effects in human neurons and astrocytes. While AA appears neurotoxic at concentrations as low as 500 nM, AA improved intracellular NAD^+^ levels in human astrocytes by up to 10% in the 0.5 to 10 μM range (Fig. 2). AA has been previously reported to impair energy metabolism in the rat cerebral cortex at micromolar concentrations, possibly through inhibition of complex I–III activities in the mitochondrial respiratory chain.^47^ While the KP is fully expressed in human neurons,^48^ kynurenine hydroxylase (KYN-OHase), which converts KYN to 3-HK is absent in human foetal astrocytes.^27^ Consequently, astrocytes cannot produce 3-HK.^27^ The KP is divided into two parts in human astrocytes (Fig. 1), and KYN may be converted to AA by kynureninase which then undergoes non-specific hydroxylation to form 3-HAA and may subsequently be used to form QUIN and NAD^+^.^27^ This suggests a greater requirement for AA as a substrate for NAD^+^ synthesis in human astrocytes compared to human neurons, and AA may become cytotoxic to both cell types in excess concentrations. These findings provide insight not only in the differences between neurons and astrocytes, but also for understanding how different brain cells produce NAD^+^.

QUIN is an endogenous NMDA receptor agonist involved in neuronal firing.^15^ The amount of QUIN in the brain and CSF is usually less than 100 nM. Increased brain QUIN levels (500–1000 nM) have been observed in the CSF and serum in several inflammatory brain diseases, including AD,^49^ HD,^45^ traumatic brain injury, AIDS dementia complex (ADC),^16^ and other infections.^15^ QUIN has been shown to be up-regulated in ageing and in association with senile plaques in the AD brain.^50^ The results in the study are in line with previously published results highlighting the importance of QUIN as a beneficial substrate for NAD^+^ synthesis at low concentration, but a putative toxin able to induce oligodendrocyte, neuronal, and astroglial apoptosis at pathophysiological concentrations.^49^,^51^–^53^

As previously mentioned, PIC is an endogenous metal chelator in the brain, and is an efficient chelator for minerals such as chromium, zinc, manganese, copper, and iron.^23^ Unbound (free) redox-active iron and copper significantly increase free radical generation and are found in abundance in the AD brain.^54^ Disordered PIC metabolism may yet be found to play a role in the pathophysiology of AD. The physiological concentration of PIC in human serum is thought to be between 100–400 nM.^55^ Elevated PIC levels have been associated with fatal outcome in Malawian children with cerebral malaria.^56^ Our data shows that PIC can increase intracellular NAD^+^ levels at physiological concentrations (100 nM). This may be partly due to its intracellular metal chelating properties reducing the contribution of free redox active metals in free radical generation.^57^

However, PIC appeared to also induce a dose dependent decrease in intracellular NAD^+^ and increase in extracellular LDH activity in both human astrocytes and neurons. These results are consistent with one study that observed signficant PIC induced neurotoxicity that was associated with both prolonged exposure time and increased glucose concentration in the culture supernatant.^41^ While the mechanism leading to PIC toxicity is not known, at high concentrations, PIC may be causing disruption to some of the many trace metal (Cu^+^, Zn^++^, Fe^++^) dependent biochemical reactions essential for normal cell metabolism.

KYNA is a neuroprotective molecule with antagonistic properties on both nicotinic-acetylcholine and glutamatergic receptors at supra-physiological concentrations.^39^ The tissue concentrations of KYNA in the human brain have been estimated to be between 0.2–1.5 μM.^58^,^59^ In our study, no significant changes in intracellular NAD^+^ levels and cell viability were observed across micromolar concentrations in both human primary astrocytes and neurons (Figs. 2 and 3). KYNA exhibits a high affinity for the glycine-binding site of the NMDA receptor at low concentrations, but can act on the glutamate binding site on the NMDA receptor, and AMPA receptors, at high micromolar concentrations.^39^ Decrease in KYNA levels below the threshold needed for NMDA receptor antagonism can enhance the vulnerability of dopaminergic neurons to excitotoxic insult.^60^ Moroni^61^ proposed that NMDA and glutamate induced excitotoxic neuronal damage can be ameliorated by diverting KYN metabolism to KYNA synthesis. Likewise, supplementation with TRP, an endogenous precursor for KYN synthesis, may increase the levels of KYNA and therefore possibly slow down or prevent the formation of cytotoxic concentrations of QUIN.^62^,^63^

This study is the first to examine the effects of extracellular KP metabolites on intracellular NAD^+^ synthesis and cell death in human primary astrocytes and neurons. While a large body of literature is available on the toxic effects of KP metabolites in regard to neuroinflammation, few studies have considered the effect of these metabolites on intracellular NAD^+^ levels *in-vitro*. This study demonstrates that changes in the brain levels of certain KP metabolites may be important mediators contributing to brain cell dysfunction in some neurological disorders involving increased KP activation.
